# Developing a nomogram-based scoring model to estimate the risk of pulmonary embolism in respiratory department patients suspected of pulmonary embolisms

**DOI:** 10.3389/fmed.2023.1164911

**Published:** 2023-05-17

**Authors:** Feng Lanfang, Ma Xu, Chen Jun, Zhao Jia, Li Wenchen, Jia Xinghua

**Affiliations:** ^1^Department of Respiratory, Affiliated Dongyang Hospital of Wenzhou Medical University, Dongyang, Zhejiang, China; ^2^Department of Vascular Surgery, Affiliated Dongyang Hospital of Wenzhou Medical University, Dongyang, Zhejiang, China; ^3^Department of Nuclear Medicine, Affiliated Dongyang Hospital of Wenzhou Medical University, Dongyang, Zhejiang, China; ^4^Operation Center, Affiliated Dongyang Hospital of Wenzhou Medical University, Dongyang, Zhejiang, China; ^5^Department of Neurology, Affiliated Dongyang Hospital of Wenzhou Medical University, Dongyang, Zhejiang, China

**Keywords:** pulmonary embolism, risk scoring tool, retrospective analysis, respiratory medicine, nomogram

## Abstract

**Objective:**

Pulmonary embolisms (PE) are clinically challenging because of their high morbidity and mortality. This study aimed to create a nomogram to accurately predict the risk of PE in respiratory department patients in order to enhance their medical treatment and management.

**Methods:**

This study utilized a retrospective method to collect information on medical history, complications, specific clinical characteristics, and laboratory biomarker results of suspected PE patients who were admitted to the respiratory department at Affiliated Dongyang Hospital of Wenzhou Medical University between January 2012 and December 2021. This study involved a total of 3,511 patients who were randomly divided into a training group (six parts) and a validation group (four parts) based on a 6:4 ratio. The LASSO regression and multivariate logistic regression were used to develop a scoring model using a nomogram. The performance of the model was evaluated using receiver operating characteristic curve (AUC), calibration curve, and clinical decision curve.

**Results:**

Our research included more than 50 features from 3,511 patients. The nomogram-based scoring model was established using six predictive features including age, smoke, temperature, systolic pressure, D-dimer, and fibrinogen, which achieved AUC values of 0.746 in the training cohort (95% CI 0.720–0.765) and 0.724 in the validation cohort (95% CI 0.695–0.753). The results of the calibration curve revealed a strong consistency between probability predicted by the nomogram and actual probability. The decision curve analysis (DCA) also demonstrated that the nomogram-based scoring model produced a favorable net clinical benefit.

**Conclusion:**

In this study, we successfully developed a novel numerical model that can predict the risk of PE in respiratory department patients suspected of PE, which can not only appropriately select PE prevention strategies but also decrease unnecessary computed tomographic pulmonary angiography (CTPA) scans and their adverse effects.

## 1. Introduction

Pulmonary embolism (PE) is the most common disease that can endanger the life of patients in respiratory and cardiovascular departments, mainly caused by various emboli blocking pulmonary arteries or branches, not only in the West, but also in China (1). It is not only related to respiratory disease but also involves cardiology, gynecology and obstetrics, oncology, hematology, surgery, and other diseases (2–6). The clinical manifestation of PE is a lack of specificity, and it is difficult to distinguish it from other respiratory diseases (3), such as chronic obstructive pulmonary disease. Its misdiagnosis rate and mortality rate are high, so it has become an important medical topic to improve the diagnosis rate and reduce the mortality of PE.

Computed tomographic pulmonary angiography (CTPA) is recommended for diagnosis and risk level assessment of PE (7–9). However, it is time consuming and expensive and even may cause serious side effects in patients. Many researchers have created a variety of risk assessment models (RAM) to predict PE, and their usability has been continuously validated. Hou et al. (10) established a novel PE risk prediction model based on machine learning (ML) methods. Robert-Ebadi et al. verified the feasibility of the simplified Geneva score in a clinic in 2017 (11). Wang et al. (12) established a novel risk assessment model to estimate the probability of PE in postoperative patients. Other studies indicated that initial blood parameters seem to enable further differentiation of patients with suspected PE and elevated d-dimers to raise pre-test probability of PE. Machine-learning-based prediction models might help to further narrow down CT indications in future (13). Other findings showed that syncope, systolic blood pressure, oxygen saturation, white blood cell, neutrophil percentage, and others, are crucial for the feature selection to assess the severity of PE (14). Kirsch et al. (15) demonstrated the ability of good score in predicting PE, which showed Wells score above 4 was significantly associated with PE but the sensitivity and specificity of the score were unreliable. Although many researchers have reported different prediction models to calculate the pretest probability of PE, they are underused and tend to underperform in practice, leading to persistent overuse of CTPA imaging for PE. Therefore, it will be convenient to have a simple and fast risk prediction model to predict the probability of PE occurrence.

Due to the non-specificity of PE in different diseases, many debates about these RAMs are presented, that there are no consensual methods to diagnose PE. In order to accurately diagnose them, it is necessary to develop a potential and appropriate model for patients in respiratory department to predict PE.

The purpose of this study was to develop and validate a numerical model based on electronic medical record (EMR) data for predicting PE in respiratory department patients.

## 2. Materials and methods

### 2.1. Study population

In this study, a total of 3,511 patients who were suspected of PE and hospitalized in the respiratory department at Affiliated Dongyang Hospital of Wenzhou Medical University from January 2012 to December 2021 were included. The data of subjects were retrospective collected from our clinical research data platform. The medical records of 3,511 subjects were statistically analyzed after the baseline data was cleared and extracted, and the subjects were randomly divided into a training cohort and a validation cohort with a 6:4 ratio.

The retrospective study was granted ethical approval by the Medical Ethics Committee of Affiliated Dongyang Hospital of Wenzhou Medical University (No: 2022-YX-160), and informed consent was waived. Patient data were processed in a confidential manner, with identifying information removed before analysis. The study was performed in accordance with the principles outlined in the Declaration of Helsinki.

### 2.2. Study outcomes and data collection

**Figure 1:**
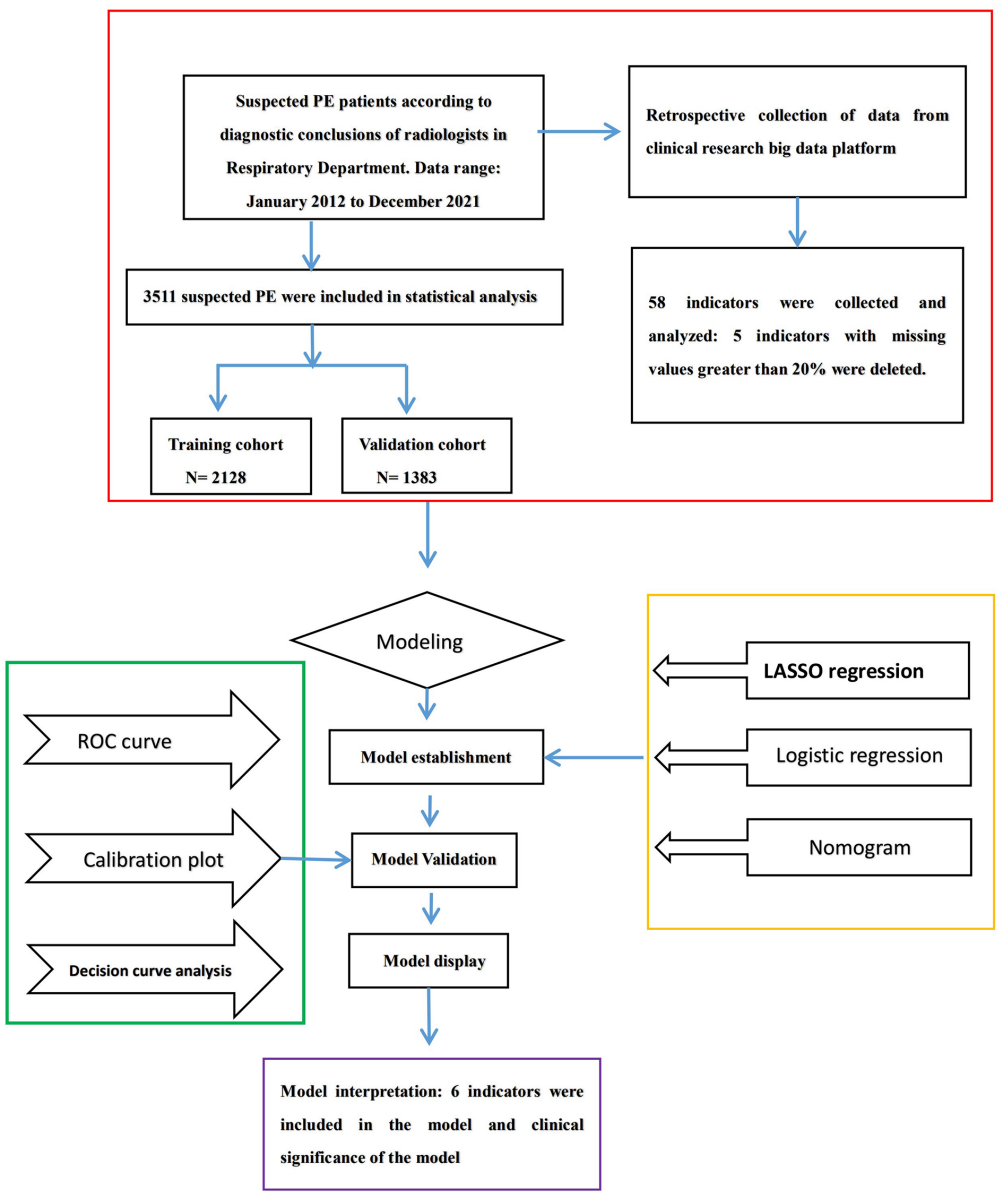
Flowchart of the processing step for predicting pulmonary embolisms (PE).

PE was defined in accordance with the criteria of European Society of Cardiology Guidelines (7). The patients who have undergone computed tomographic pulmonary angiography (CTPA) examination are suspected of PE. PE was confirmed by an identified filling defect in the pulmonary artery system in CTPA, including subsegmental PE. Based on the diagnostic conclusions of radiologists, we used this to determine whether PE occurred. The past medical history, complications, individual clinical features, and clinic biomarkers data were collected. Our research flow chart is shown in Figure 1.

### 2.3. Statistical analysis

The data were analyzed using R Studio software for Windows. Continuous variables were expressed as the mean with standard deviation (SD) or median with interquartile range (IQR) and compared using Student’s *t*-test or the Mann–Whitney *U*-test. Categorical variables were expressed as frequency with percentage and compared using the chi-squared test or Fisher’s exact test. All subjects contain 58 variables. To guarantee the reliability of the data, five indicators with missing values greater than 20% were deleted. Use the (mice) package in R software for multiple imputation techniques (16) which were utilized to impute the remaining missing values of the predictor variables. Use the (glmnet) package for the least absolute shrinkage and selection operator (LASSO) analysis which regression model (17–19) was performed to tackle the collinearity of candidate variables to select the optimal predictive variables. The results were expressed as odds ratios (ORs) and 95% confidence intervals (CIs). A two-tailed *p*-value <0.05 was considered to be statistically significant.

### 2.4. Model development and validation, and evaluation

By combining the selected predictors from the LASSO analysis, the (rms) package for multivariable logistic regression analysis was performed to develop the PE prediction model. Use the (regplot) package in R software for nomogram. The performance of the model was evaluated in terms of its discrimination, calibration, and clinical utility (20). The performance of the model was assessed using various evaluation metrics, including the (pROC) package for the area under the receiver operating characteristic (ROC) curve (AUC), using the (calibrate) package for calibration curve, and using the (rmda) package for decision curve analysis (DCA). The AUC measured the discrimination capability of the model, while the calibration curve evaluated the consistency between the predicted and actual probabilities of PE. The DCA was used to quantify the net clinical benefits at different thresholds.

## 3. Results

### 3.1. Characteristics of the study population

A total of five variables were removed from our data due to excessive amounts of missing information (over 20%) before proceeding with the analysis. We involved 53 variables in which missing data of less than 20% can be found in this study (shown in “Appendix 1”). The data contained missing information for 53 variables, with a range of missing data percentage from 0.00%–19.74%. Multiple imputation techniques were used to complete the missing data. A total of 3,511 subjects suspected of PE were included in the present study, with an incidence rate of 28.36%. The demographic and clinical characteristics of these patients are displayed in Table 1. The sample was randomly divided into two groups, the training cohort (*n* = 2,128) and the validation cohort (*n* = 1,383), and their respective characteristics are summarized in Table 2.

**Table 1:** Baseline characteristics of subjects.

Variables	Total (*n* = 3,511)	No PE (*n* = 2,515)	PE (*n* = 996)	*p*
Gender, *n* (%)				0.103
Female	1,442 (41.1)	1,011 (40.2)	431 (43.3)	
Male	2069 (58.9)	1,504 (59.8)	565 (56.7)	
Age (years), median (*Q*1, *Q*3)	73 (63, 81)	72 (62, 80)	75 (66, 82)	<0.001
Temperature (°C), median (*Q*1, *Q*3)	37.4 (37, 38.2)	37.4 (36.9, 38.2)	37.4 (37, 38.1)	0.953
Breathing (/min), median (*Q*1, *Q*3)	24 (21, 28)	22 (20, 26)	24 (22, 28)	<0.001
Pulse (/min), median (*Q*1, *Q*3)	106 (91, 120)	104 (89, 119.5)	110 (98, 123)	<0.001
Systolic pressure (mmHg), median (*Q*1, *Q*3)	101 (91, 116)	104 (93, 120)	95 (89, 106)	<0.001
Diastolic pressure (mmHg), median (*Q*1, *Q*3)	55 (48, 66)	56 (49, 69)	52 (46, 59)	<0.001
Headache, *n* (%)				0.003
No	3,335 (95)	2,371 (94.3)	964 (96.8)	
Yes	176 (5)	144 (5.7)	32 (3.2)	
Dizzy, *n* (%)				0.691
No	3,372 (96)	2,418 (96.1)	954 (95.8)	
Yes	139 (4)	97 (3.9)	42 (4.2)	
Chest tightness, *n* (%)				0.431
No	1907 (54.3)	1,377 (54.8)	530 (53.2)	
Yes	1,604 (45.7)	1,138 (45.2)	466 (46.8)	
Anhelation, *n* (%)				0.271
No	2041 (58.1)	1,477 (58.7)	564 (56.6)	
Yes	1,470 (41.9)	1,038 (41.3)	432 (43.4)	
Hemoptysis, *n* (%)				0.014
No	3,315 (94.4)	2,359 (93.8)	956 (96)	
Yes	196 (5.6)	156 (6.2)	40 (4)	
Chest pain, *n* (%)				0.415
No	3,356 (95.6)	2,399 (95.4)	957 (96.1)	
Yes	155 (4.4)	116 (4.6)	39 (3.9)	
Syncope, *n* (%)				<0.001
No	3,443 (98.1)	2,479 (98.6)	964 (96.8)	
Yes	68 (1.9)	36 (1.4)	32 (3.2)	
Cough, *n* (%)				<0.001
No	1833 (52.2)	1,268 (50.4)	565 (56.7)	
Yes	1,678 (47.8)	1,247 (49.6)	431 (43.3)	
Fever, *n* (%)				0.003
0	3,149 (89.7)	2,231 (88.7)	918 (92.2)	
1	362 (10.3)	284 (11.3)	78 (7.8)	
Lower limb edema, *n* (%)				0.184
No	3,296 (93.9)	2,370 (94.2)	926 (93)	
Yes	215 (6.1)	145 (5.8)	70 (7)	
COPD, *n* (%)				0.279
No	1858 (52.9)	1,316 (52.3)	542 (54.4)	
Yes	1,653 (47.1)	1,199 (47.7)	454 (45.6)	
Hypertension, *n* (%)				0.133
No	2070 (59)	1,503 (59.8)	567 (56.9)	
Yes	1,441 (41)	1,012 (40.2)	429 (43.1)	
Diabetes, *n* (%)				0.272
No	3,150 (89.7)	2,247 (89.3)	903 (90.7)	
Yes	361 (10.3)	268 (10.7)	93 (9.3)	
Coronary heart disease, *n* (%)				0.402
No	2,344 (66.8)	1,668 (66.3)	676 (67.9)	
Yes	1,167 (33.2)	847 (33.7)	320 (32.1)	
Hyperlip, *n* (%)				0.829
No	3,461 (98.6)	2,478 (98.5)	983 (98.7)	
Yes	50 (1.4)	37 (1.5)	13 (1.3)	
Atrial fibrillation, *n* (%)				<0.001
No	3,218 (91.7)	2,333 (92.8)	885 (88.9)	
Yes	293 (8.3)	182 (7.2)	111 (11.1)	
Operation, *n* (%)				0.836
No	3,493 (99.5)	2,503 (99.5)	990 (99.4)	
Yes	18 (0.5)	12 (0.5)	6 (0.6)	
Tumor, *n* (%)				0.064
No	3,142 (89.5)	2,235 (88.9)	907 (91.1)	
Yes	369 (10.5)	280 (11.1)	89 (8.9)	
Smoke, *n* (%)				<0.001
No	2,138 (60.9)	1,483 (59)	655 (65.8)	
Yes	1,373 (39.1)	1,032 (41)	341 (34.2)	
Drink, *n* (%)				0.264
No	2,385 (67.9)	1,694 (67.4)	691 (69.4)	
Yes	1,126 (32.1)	821 (32.6)	305 (30.6)	
WBC (10^9^/L), median (*Q*1, *Q*3)	7.92 (5.91, 10.96)	7.74 (5.75, 10.79)	8.26 (6.25, 11.3)	<0.001
Lactate (mmol/L), median (*Q*1, *Q*3)	1.5 (1.1, 2)	1.5 (1.1, 2)	1.5 (1.1, 2.1)	<0.001
RBC (10^12^/L), median (*Q*1, *Q*3)	4.31 (3.95, 4.68)	4.33 (3.97, 4.7)	4.27 (3.9, 4.65)	0.01
Mg (mmol/L), median (*Q*1, *Q*3)	0.89 (0.83, 0.95)	0.89 (0.84, 0.95)	0.89 (0.83, 0.94)	0.016
HGB (g/L), median (*Q*1, *Q*3)	131 (119, 143)	131 (119, 143)	130 (116, 141.25)	0.001
Hct, Median (*Q*1, *Q*3)	0.4 (0.36, 0.43)	0.4 (0.36, 0.43)	0.39 (0.36, 0.43)	0.03
Neutrophi percent, median (*Q*1, *Q*3)	0.78 (0.68, 0.86)	0.77 (0.67, 0.86)	0.8 (0.71, 0.87)	<0.001
Neutrophil count (10^9^/L), median (*Q*1, *Q*3)	5.92 (3.98, 8.98)	5.7 (3.85, 8.87)	6.53 (4.41, 9.27)	<0.001
Lymphocyte percent, median (*Q*1, *Q*3)	0.21 (0.14, 0.28)	0.21 (0.14, 0.29)	0.19 (0.13, 0.27)	<0.001
Lymphocyte count (10^9^/L), median (*Q*1, *Q*3)	1.33 (0.97, 1.8)	1.34 (0.99, 1.82)	1.31 (0.93, 1.76)	0.028
PLT (10^9^/L), median (*Q*1, *Q*3)	217 (171, 275)	219 (172.5, 274)	213.5 (169, 275)	0.514
ALB (g/L), median (*Q*1, *Q*3)	36.6 (32.9, 40.1)	37 (33.2, 40.3)	35.8 (32.1, 39.4)	<0.001
PDW(%), median (*Q*1, *Q*3)	15.9 (13.1, 16.3)	15.9 (12.7, 16.3)	16 (14.7, 16.4)	<0.001
RDW (%), median (*Q*1, *Q*3)	0.13 (0.13, 0.14)	0.13 (0.12, 0.14)	0.13 (0.13, 0.14)	<0.001
HDL (mmol/L), median (*Q*1, *Q*3)	1.06 (0.87, 1.29)	1.06 (0.87, 1.3)	1.04 (0.86, 1.28)	0.382
LDL (mmol/L), median (*Q*1, *Q*3)	2.34 (1.84, 2.88)	2.34 (1.84, 2.86)	2.32 (1.83, 2.91)	0.91
Apolipoprotein A1(g/L), median (*Q*1, *Q*3)	1.01 (0.83, 1.24)	1.02 (0.84, 1.24)	0.99 (0.82, 1.21)	0.035
Apolipoprotein B (g/L), median (*Q*1, *Q*3)	0.82 (0.66, 0.99)	0.82 (0.66, 0.99)	0.83 (0.66, 0.99)	0.587
TG (mmol/L), median (*Q*1, *Q*3)	1.1 (0.83, 1.51)	1.1 (0.83, 1.53)	1.1 (0.84, 1.47)	0.958
TC (mmol/L), median (*Q*1, *Q*3)	4.06 (3.45, 4.71)	4.06 (3.46, 4.71)	4.03 (3.42, 4.72)	0.288
Fibrinogen (g/L), median (*Q*1, *Q*3)	4.3 (3.27, 5.76)	4.31 (3.24, 5.86)	4.28 (3.34, 5.53)	0.451
D-dimer (mg/L), median (*Q*1, *Q*3)	1.31 (0.73, 3.6)	1.1 (0.66, 2.37)	3.18 (1.16, 7.99)	<0.001
PT (s), median (*Q*1, *Q*3)	13.8 (13.1, 14.6)	13.7 (13.1, 14.5)	14.1 (13.4, 14.9)	<0.001
APTT (s), median (*Q*1, *Q*3)	38.9 (35.7, 43.3)	39.1 (35.8, 43.3)	38.6 (35.4, 43.4)	0.247
TT (s), median (*Q*1, *Q*3)	16.3 (15.6, 17)	16.3 (15.6, 17)	16.3 (15.6, 17.1)	0.082
pro.BNP (pg/mL), median (*Q*1, *Q*3)	377.9 (106.35, 1429.1)	281.5 (86.15, 1078.45)	830.65 (217.9, 2029.25)	<0.001

WBC, white blood cell count; RBC, red blood cell count; Mg, magnesium; HGB, hemoglobin; Hct, hematocrit; PLT, platelet count; ALB, albumin; PDW, platelet distribution width; RDW, red blood cell distribution width; HDL, high-density lipoprotein; LDL, low-density lipoprotein; TG, triglyceride; TC, total cholesterol; PT, prothrombin time; APTT, activated partial prothrombin time; TT, thrombin time; pro.BNP, B-type natriuretic peptide precursor.

**Table 2:** Baseline characteristics of the enrolled patients in the training and validation cohort.

Variables	Total (*n* = 3,511)	Training (*n* = 2,128)	Validation (*n* = 1,383)	*p*
PE, *n* (%)				0.692
No	2,515 (71.6)	1,530 (71.9)	985 (71.2)	
Yes	996 (28.4)	598 (28.1)	398 (28.8)	
Gender, *n* (%)				0.461
Female	1,442 (41.1)	885 (41.6)	557 (40.3)	
Male	2069 (58.9)	1,243 (58.4)	826 (59.7)	
Age (years), median (*Q*1, *Q*3)	73 (63, 81)	72 (63, 80)	73 (64, 81)	0.177
Temperature (°C), median (*Q*1, *Q*3)	37.4 (37, 38.2)	37.4 (37, 38.1)	37.4 (36.9, 38.2)	0.706
Breathing (/min), median (*Q*1, *Q*3)	24 (21, 28)	24 (21, 26.25)	24 (22, 28)	0.016
Pulse (/min), median (*Q*1, *Q*3)	106 (91, 120)	105 (90, 120)	106 (92, 121)	0.262
Systolic pressure (mmHg), median (*Q*1, *Q*3)	101 (91, 116)	101 (91, 116)	101 (91, 117)	0.859
Diastolic pressure (mmHg), median (*Q*1, *Q*3)	55 (48, 66)	55 (48, 65)	55 (47, 67)	0.949
Headache, *n* (%)				0.054
No	3,335 (95)	2034 (95.6)	1,301 (94.1)	
Yes	176 (5)	94 (4.4)	82 (5.9)	
Dizzy, *n* (%)				1
No	3,372 (96)	2044 (96.1)	1,328 (96)	
Yes	139 (4)	84 (3.9)	55 (4)	
Chest tightness, *n* (%)				0.133
No	1907 (54.3)	1,178 (55.4)	729 (52.7)	
Yes	1,604 (45.7)	950 (44.6)	654 (47.3)	
Anhelation, *n* (%)				0.383
No	2041 (58.1)	1,250 (58.7)	791 (57.2)	
Yes	1,470 (41.9)	878 (41.3)	592 (42.8)	
Hemoptysis, *n* (%)				0.684
No	3,315 (94.4)	2006 (94.3)	1,309 (94.6)	
Yes	196 (5.6)	122 (5.7)	74 (5.4)	
Chest pain, *n* (%)				0.211
No	3,356 (95.6)	2042 (96)	1,314 (95)	
Yes	155 (4.4)	86 (4)	69 (5)	
Syncope, *n* (%)				0.152
No	3,443 (98.1)	2093 (98.4)	1,350 (97.6)	
Yes	68 (1.9)	35 (1.6)	33 (2.4)	
Cough, *n* (%)				0.226
No	1833 (52.2)	1,129 (53.1)	704 (50.9)	
Yes	1,678 (47.8)	999 (46.9)	679 (49.1)	
Fever, *n* (%)				0.208
No	3,149 (89.7)	1897 (89.1)	1,252 (90.5)	
Yes	362 (10.3)	231 (10.9)	131 (9.5)	
Lower limb edema, *n* (%)				0.978
No	3,296 (93.9)	1997 (93.8)	1,299 (93.9)	
Yes	215 (6.1)	131 (6.2)	84 (6.1)	
COPD, *n* (%)				0.61
No	1858 (52.9)	1,134 (53.3)	724 (52.3)	
Yes	1,653 (47.1)	994 (46.7)	659 (47.7)	
Hypertension, *n* (%)				0.33
No	2070 (59)	1,269 (59.6)	801 (57.9)	
Yes	1,441 (41)	859 (40.4)	582 (42.1)	
Diabetes, *n* (%)				0.517
No	3,150 (89.7)	1903 (89.4)	1,247 (90.2)	
Yes	361 (10.3)	225 (10.6)	136 (9.8)	
Coronary heart disease, *n* (%)				0.386
No	2,344 (66.8)	1,433 (67.3)	911 (65.9)	
Yes	1,167 (33.2)	695 (32.7)	472 (34.1)	
Hyperlip, *n* (%)				0.522
No	3,461 (98.6)	2095 (98.4)	1,366 (98.8)	
Yes	50 (1.4)	33 (1.6)	17 (1.2)	
Atrial fibrillation, *n* (%)				0.991
No	3,218 (91.7)	1951 (91.7)	1,267 (91.6)	
Yes	293 (8.3)	177 (8.3)	116 (8.4)	
Operation, *n* (%)				0.495
No	3,493 (99.5)	2,119 (99.6)	1,374 (99.3)	
Yes	18 (0.5)	9 (0.4)	9 (0.7)	
Tumor, *n* (%)				0.111
No	3,142 (89.5)	1919 (90.2)	1,223 (88.4)	
Yes	369 (10.5)	209 (9.8)	160 (11.6)	
Smoke, *n* (%)				0.814
No	2,138 (60.9)	1,292 (60.7)	846 (61.2)	
Yes	1,373 (39.1)	836 (39.3)	537 (38.8)	
Drink, *n* (%)				0.103
No	2,385 (67.9)	1,423 (66.9)	962 (69.6)	
Yes	1,126 (32.1)	705 (33.1)	421 (30.4)	
WBC (10^9^/L), median (*Q*1, *Q*3)	7.92 (5.91, 10.96)	8.04 (5.98, 11)	7.72 (5.73, 10.94)	0.075
Lactate (mmol/), median (*Q*1, *Q*3)	1.5 (1.1, 2)	1.5 (1.1, 2)	1.5 (1.1, 2.1)	0.037
RBC (10^12^/L), median (*Q*1, *Q*3)	4.31 (3.95, 4.68)	4.33 (3.96, 4.69)	4.29 (3.94, 4.68)	0.147
Mg (mmol/L), median (*Q*1, *Q*3)	0.89 (0.83, 0.95)	0.89 (0.83, 0.94)	0.9 (0.84, 0.95)	0.241
HGB (g/L), median (*Q*1, *Q*3)	131 (119, 143)	131 (119, 143)	130 (118, 142)	0.08
Hct, Median (*Q*1, *Q*3)	0.4 (0.36, 0.43)	0.4 (0.36, 0.43)	0.39 (0.36, 0.43)	0.03
Neutrophi percent, median (*Q*1, *Q*3)	0.78 (0.68, 0.86)	0.78 (0.68, 0.86)	0.78 (0.68, 0.87)	0.994
Neutrophil count (10^9^/L), median (*Q*1, *Q*3)	5.92 (3.98, 8.98)	6.03 (4.04, 9.06)	5.79 (3.89, 8.91)	0.227
Lymphocyte percent, median (*Q*1, *Q*3)	0.21 (0.14, 0.28)	0.2 (0.14, 0.29)	0.21 (0.13, 0.28)	0.511
Lymphocyte count (10^9^/L), median (*Q*1, *Q*3)	1.33 (0.97, 1.8)	1.35 (0.98, 1.82)	1.29 (0.95, 1.77)	0.041
PLT (10^9^/L), median (*Q*1, *Q*3)	217 (171, 275)	217 (172, 274)	216 (169, 276)	0.691
ALB (g/L), median (*Q*1, *Q*3)	36.6 (32.9, 40.1)	36.8 (32.8, 40.1)	36.5 (33, 40.1)	0.907
PDW (%), median (*Q*1, *Q*3)	15.9 (13.1, 16.3)	15.9 (13.2, 16.3)	15.9 (12.7, 16.3)	0.408
RDW (%), median (*Q*1, *Q*3)	0.13 (0.13, 0.14)	0.13 (0.13, 0.14)	0.13 (0.13, 0.14)	0.493
HDL (mmol/L), median (*Q*1, *Q*3)	1.06 (0.87, 1.29)	1.06 (0.87, 1.29)	1.05 (0.86, 1.29)	0.636
LDL (mmol/L), median (*Q*1, *Q*3)	2.34 (1.84, 2.88)	2.34 (1.85, 2.9)	2.33 (1.81, 2.83)	0.467
Apolipoprotein A1(g/L), median (*Q*1, *Q*3)	1.01 (0.83, 1.24)	1.02 (0.84, 1.24)	1 (0.82, 1.22)	0.085
Apolipoprotein B (g/L), median (*Q*1, *Q*3)	0.82 (0.66, 0.99)	0.82 (0.66, 0.99)	0.82 (0.66, 0.98)	0.716
TG (mmol/L), median (*Q*1, *Q*3)	1.1 (0.83, 1.51)	1.1 (0.84, 1.49)	1.1 (0.83, 1.54)	0.873
TC (mmol/L), median (*Q*1, *Q*3)	4.06 (3.45, 4.71)	4.06 (3.46, 4.69)	4.06 (3.45, 4.73)	0.606
Fibrinogen (g/L), median (*Q*1, *Q*3)	4.3 (3.27, 5.76)	4.31 (3.25, 5.81)	4.29 (3.28, 5.68)	0.974
D-dimer (mg/L), median (*Q*1, *Q*3)	1.31 (0.73, 3.6)	1.29 (0.73, 3.56)	1.35 (0.73, 3.7)	0.404
PT (s), median (*Q*1, *Q*3)	13.8 (13.1, 14.6)	13.8 (13.1, 14.6)	13.9 (13.2, 14.7)	0.165
APTT (s), median (*Q*1, *Q*3)	38.9 (35.7, 43.3)	38.8 (35.7, 43.5)	39 (35.6, 43)	0.464
TT (s), median (*Q*1, *Q*3)	16.3 (15.6, 17)	16.3 (15.6, 17.1)	16.3 (15.6, 17)	0.261
pro.BNP (pg/mL), median (*Q*1, *Q*3)	377.9 (106.35, 1429.1)	357.25 (104.83, 1351.25)	408.5 (108.4, 1,503)	0.112

WBC, white blood cell count; RBC, red blood cell count; Mg, magnesium; HGB, hemoglobin; Hct, hematocrit; PLT, platelet count; ALB, albumin; PDW, platelet distribution width; RDW, red blood cell distribution width; HDL, high-density lipoprotein; LDL, low-density lipoprotein; TG, triglyceride; TC, total cholesterol; PT, prothrombin time; APTT, activated partial prothrombin time; TT, thrombin time; pro.BNP, B-type natriuretic peptide precursor.

### 3.2. Selected predictors

**Figure 2:**
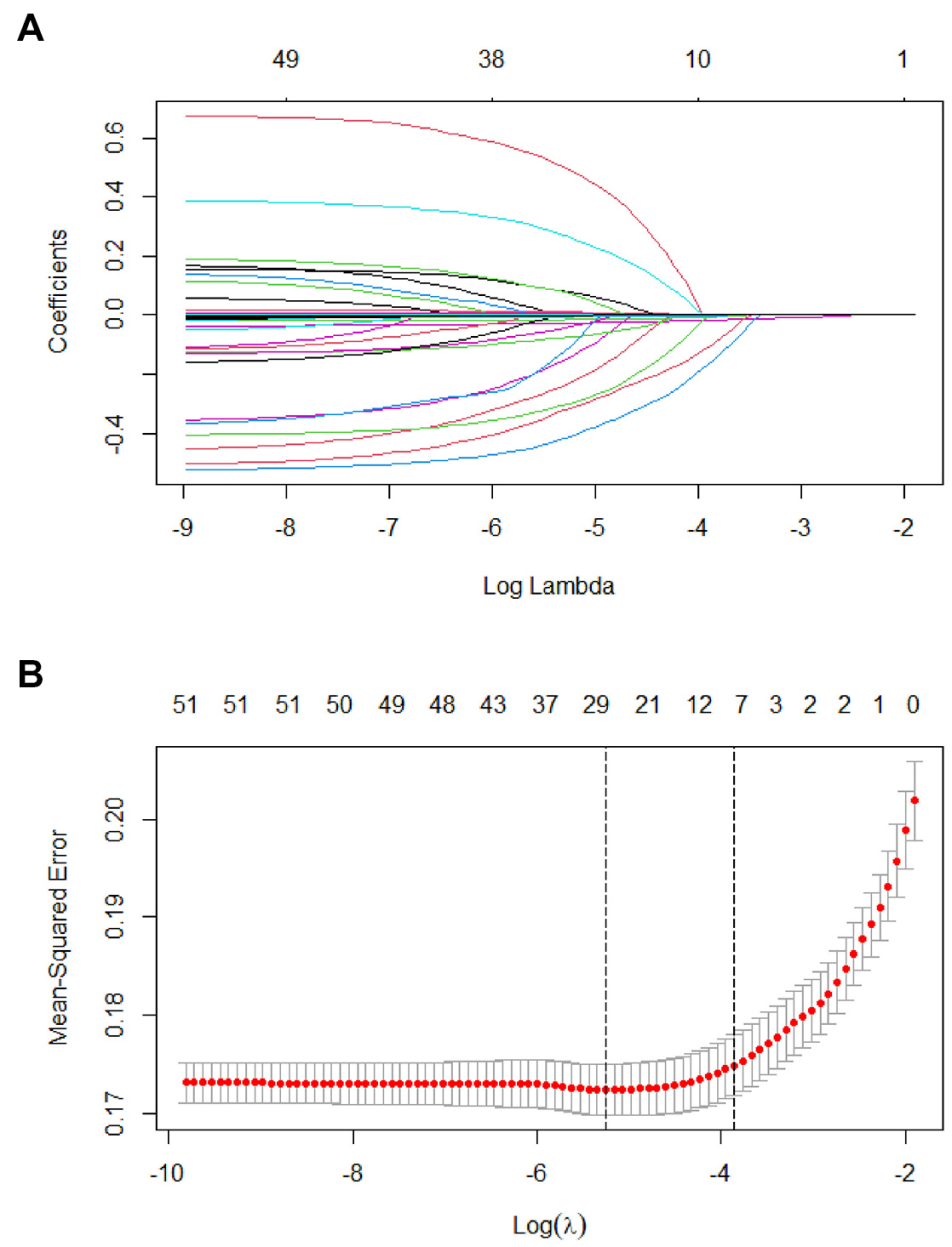
Tuning parameter selection using the LASSO regression in the training cohort. **(A)** LASSO coefficient profiles of the clinical features. **(B)** Optimal penalization coefficient lambda was generated in the LASSO via 10-fold cross validation. The lambda value of the one-fold mean square error for the training cohort.

In 53 variables, six potential predictors were finally selected on the basis of LASSO regression analysis (Figures 2A,B). The optimal predictors incorporated age, temperature, systolic pressure, fibrinogen, D-dimer, and smoke. The final predictive model was generated through a multivariable logistic regression analysis incorporating six predictors that were selected from the LASSO regression analysis (presented in Table 3).

**Table 3:** Final model coefficients.

Variables	*β*	SE	OR	95%CI	*p*
Age	0.013	0.004	1.01	1.01–1.02	0.001
Temperature	−0.204	0.067	0.82	0.72–0.93	0.002
Systolic_pressure	−0.028	0.003	0.97	0.97–0.98	<0.001
Fibrinogen	−0.101	0.031	0.9	0.85–0.96	0.001
D-dimer	0.122	0.012	1.13	1.1–1.16	<0.001
Smoke	−0.416	0.11	0.66	0.53–0.82	<0.001

### 3.3. Construction and validation of the model

**Figure 3:**
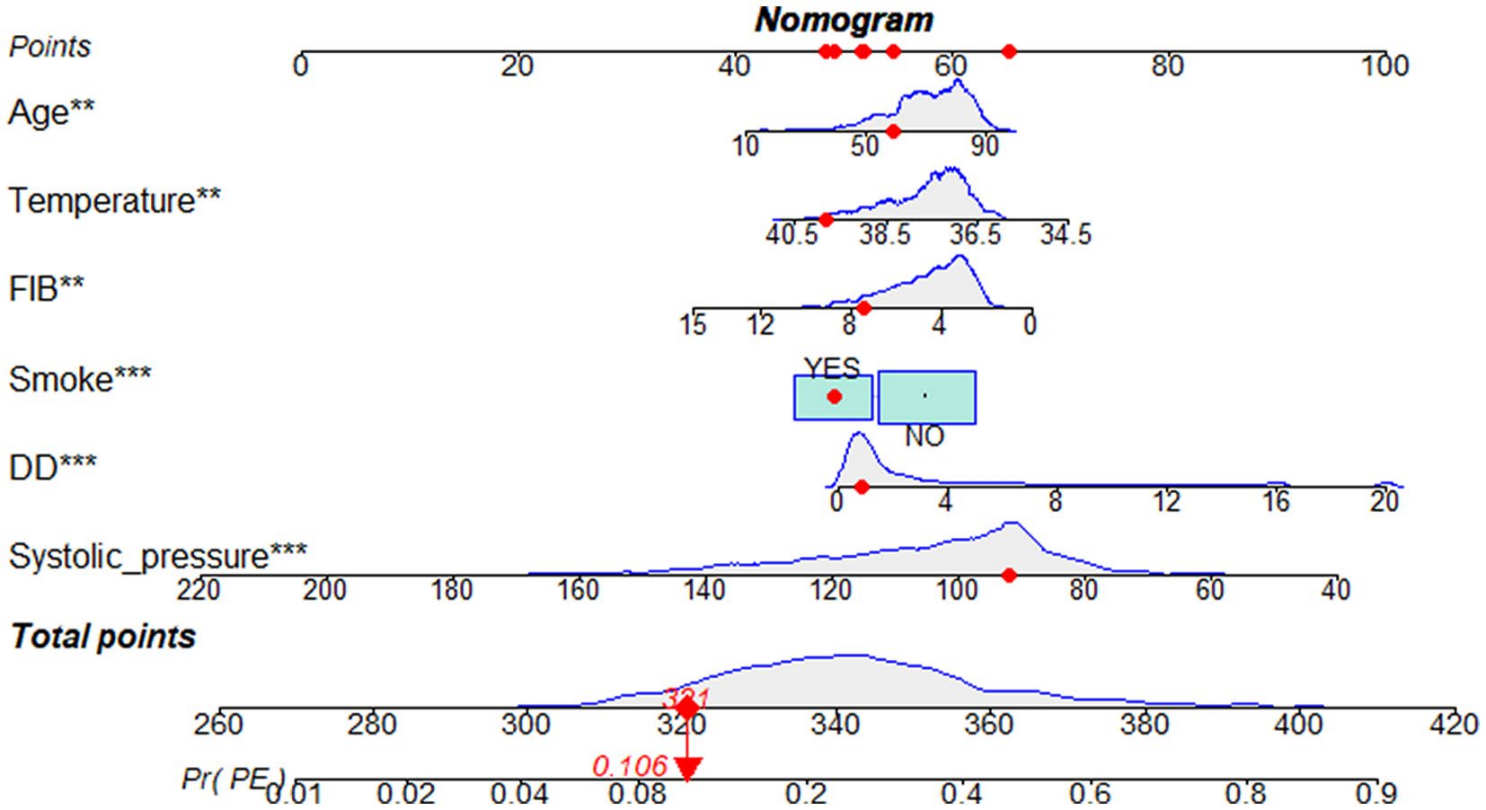
Nomogram based on the combination of six indicators was developed using logistic regression analysis. If a patient with the total score is 321, then the probability of the PE is 0.106 (red numbers). FIB, fibrinogen; DD, D-dimer.

**Figure 4:**
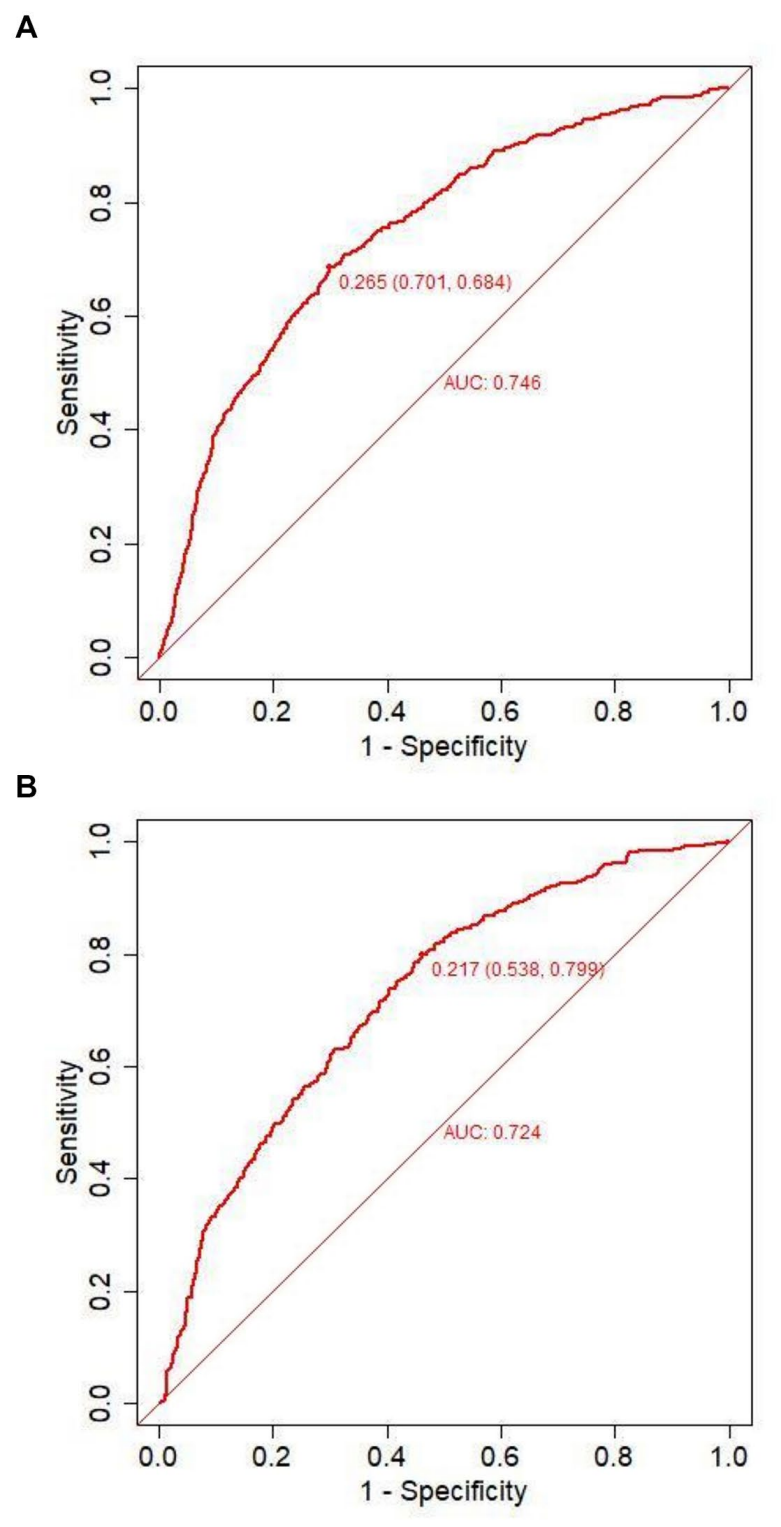
Receiver operating characteristic curves of the model distinguishing PE from non-PE in the training **(A)** and validation **(B)** cohort.

**Figure 5:**
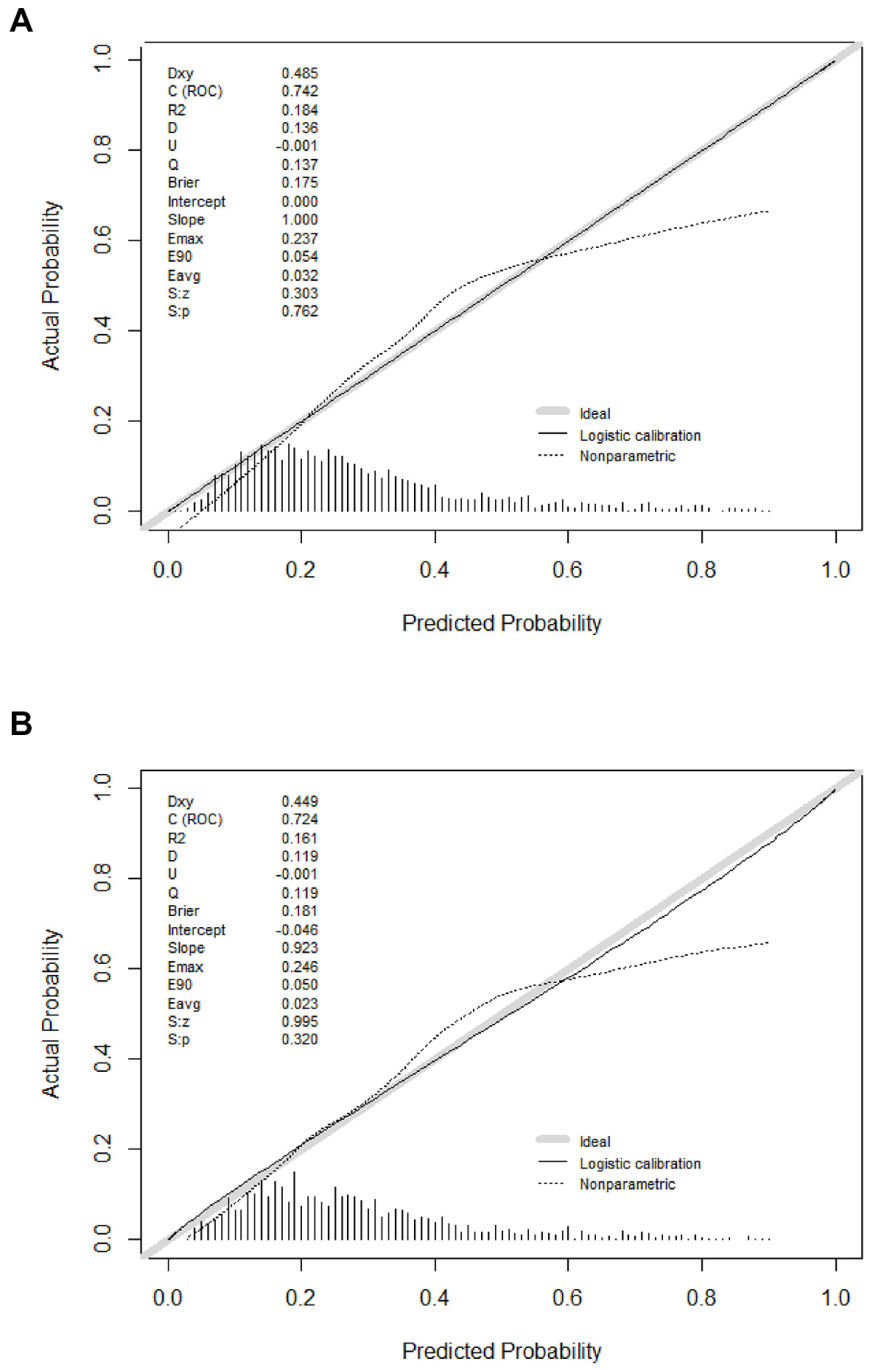
Calibration curves of the model in the training **(A)** and validation **(B)** cohort.

**Figure 6:**
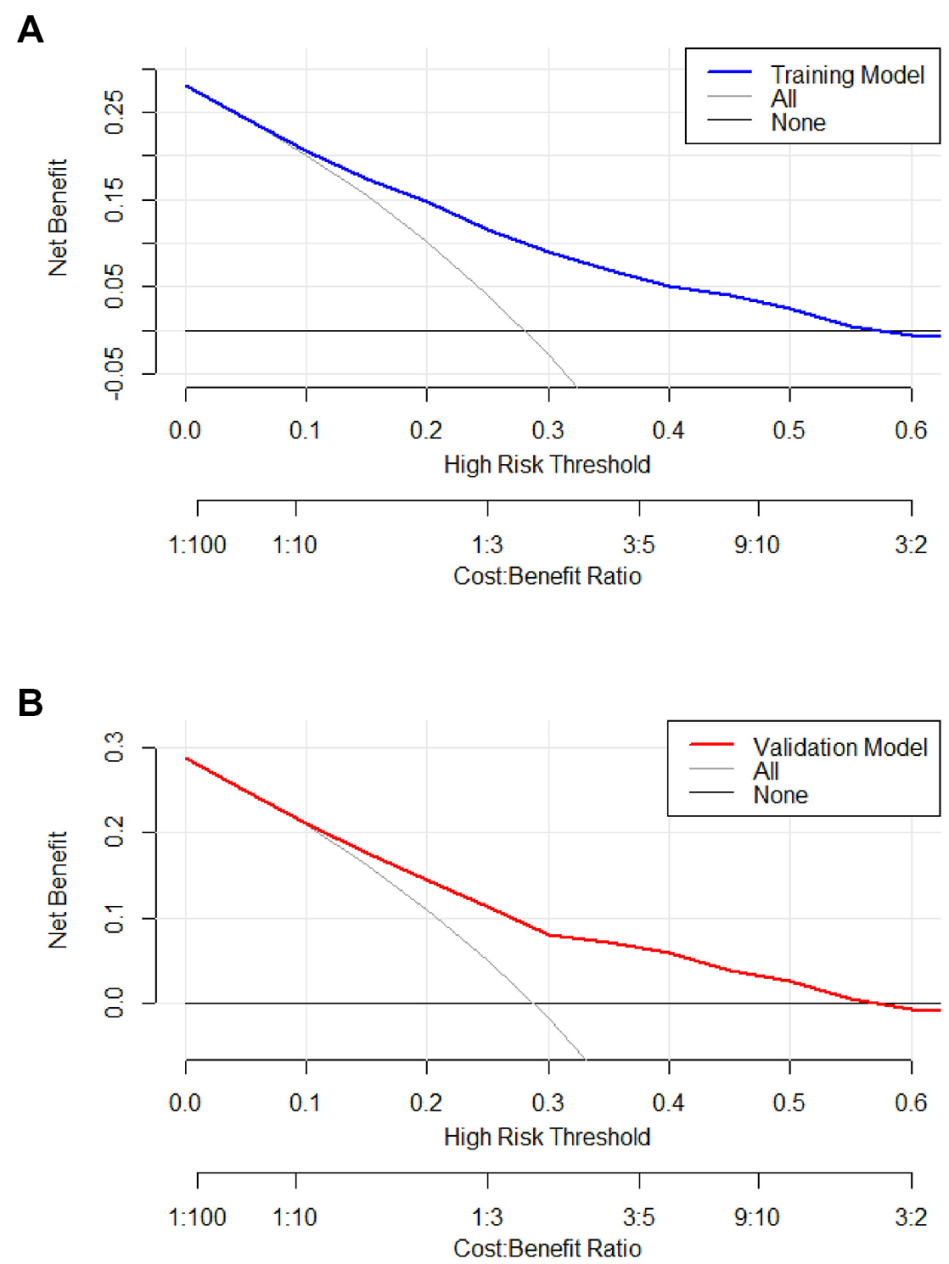
Decision curve of the model in the training **(A)** and validation **(B)** cohort. If the risk threshold is less than 60%, the nomogram model will obtain more benefit than all treatment (assuming all respiratory department patients were PE) or no treatment (assuming all respiratory department patients were non-PE).

Predicting model for PE was visualized by a nomogram, which is shown in Figure 3. The discrimination of the model, as measured by the area under the receiver operating characteristic curve (AUC), was found to be 0.746 (95% CI 0.720–0.765) in the training cohort and 0.724 (95% CI 0.695–0.753) in the validation cohort. This suggests that the predictive model has the ability to effectively differentiate PE from non-PE (Figures 4A,B). The calibration plot in training and validation cohorts (Figures 5A,B) demonstrates good consistency between PE discriminated by the model and the actual occurrence PE. The clinical utility of the nomogram was evaluated through decision curve analysis, which assessed the net benefit and threshold probabilities (shown in Figures 6A,B), indicating that the nomogram had a favorable net benefit across a broad range of threshold probabilities for PE patients in the respiratory department in both the training and validation cohorts.

### 3.4. Comparison of model discrimination ability

**Figure 7:**
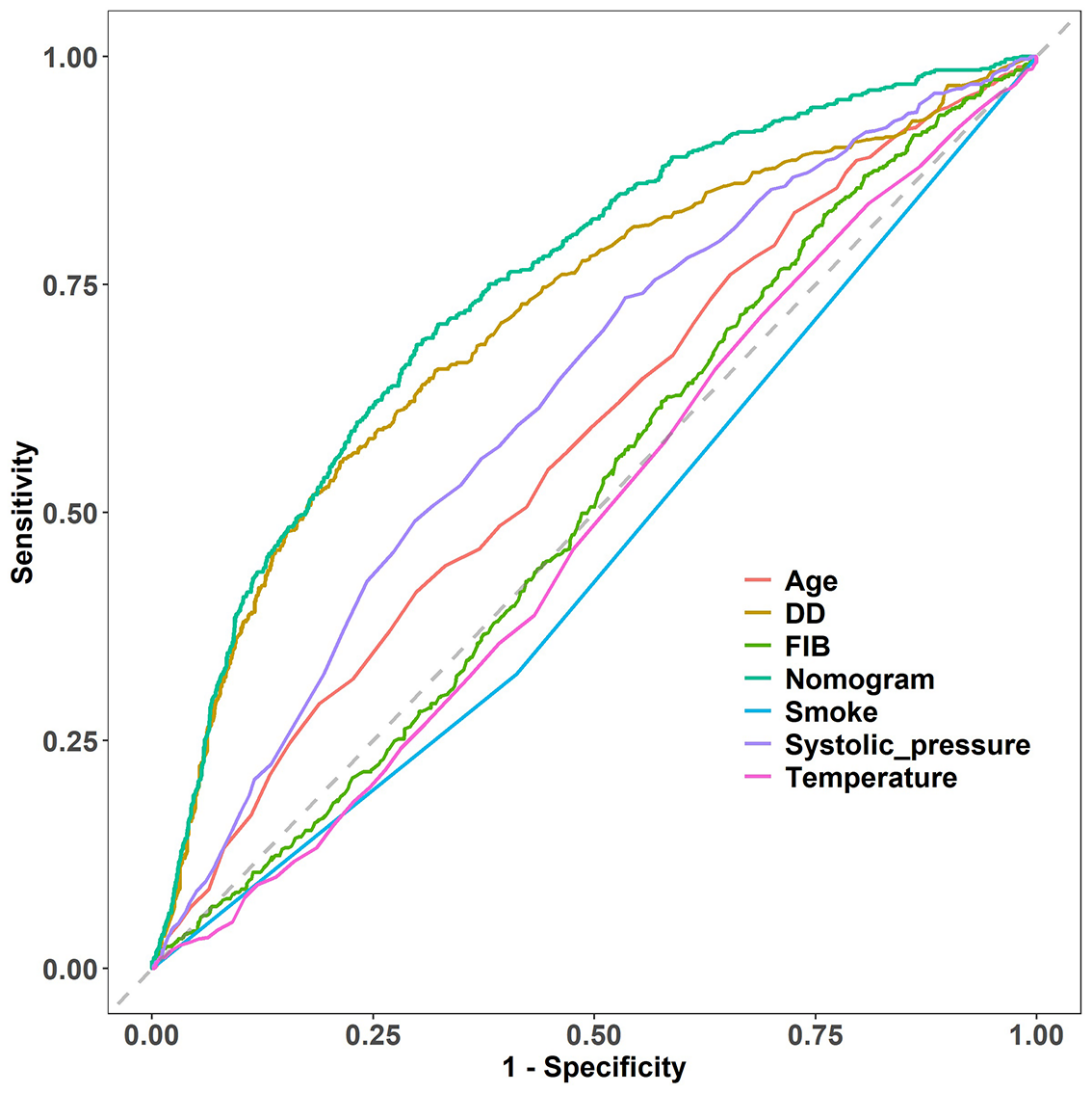
Comparison of model discrimination ability (FIB, fibrinogen; DD, D-dimer).

The performance of the nomogram was demonstrated by its AUC of 0.746 (95% CI 0.720–0.765), compared to the discriminative ability of the models which revealed that the nomogram model exhibited superior accuracy in the prediction of clinical outcomes than individual indicators (age, temperature, systolic pressure, fibrinogen, and D-dimer), as shown in Figure 7.

## 4. Discussion

The purpose of this study was to develop and validate a numerical model based on patient electronic medical record (EMR) data for predicting PE in respiratory department patients. In this study, a novel numerical model was developed incorporating six predictor variables, namely, age, temperature, systolic pressure, fibrinogen, D-dimer, and smoke. All parameters are readily available clinical features and biomarkers in routine health examinations. Notably, receiver operating characteristic (ROC) analysis indicated that our model AUC was 0.746 (95% CI 0.720–0.765) and displayed good discrimination and calibration. The decision curve analysis and clinical impact curve demonstrated that the majority of the threshold probabilities in this model exhibited favorable net benefits.

Our findings indicated that an increase in D-dimer levels was associated with a higher incidence of PE (OR 1.13; 95% CI 1.10–1.16). This result is in accordance with earlier observations (21, 22), which showed that high D-dimer level attributable to the possibility of developing PE. In terms of biomarkers, D-dimer is the only biomarker currently used in routine practice to predict PE, but it is unlikely to have adequate specificity in respiratory department patients at conventional thresholds for positivity. A large sample study from 2000 to 2015 showed increased hospitalization rates and the highest inpatient mortality for PE in elderly patients (23). A retrospective study demonstrated an association between age and the severity of the submassive PE stadium (24). Our model also shows that age (OR 1.01; 95% CI 1.01–1.02) is a high-risk factor for PE, which is similar to previous studies. In our model, two factors were positively associated with the risk of PE, whereas temperature, systolic pressure, fibrinogen, and smoke were negatively associated. A previous study showed a low systolic pressure was connected with a raised risk of PE-related mortality (25, 26). Fibrinogen is a large, complex, fibrous glycoprotein, which is converted into fibrin during the coagulation cascade, yielding the fibrin clot for hemostasis. Additionally, it was discovered that fibrinogen is synthesized as an acute phase reactant by the liver in response to inflammation or ischemia (27). A prospective study assessed the D-dimer and fibrinogen levels in 191 outpatients with suspected PE and observed that patients suffering from PE had a lower fibrinogen and higher D-dimer/fibrinogen (D/F) ratio versus those without PE (28). Our findings corroborated the inverse correlation between D-dimer and fibrinogen, which suggests that heightened coagulation prompts the consumption of fibrinogen and activation of endogenous fibrinolysis, leading to a rise in D-dimer levels. Our data also revealed that two clinical indicators including body temperature and smoke were incorporated into the model to predict PE although smoking has little effect on the whole model. Relevant studies (29) also show that fever and smoke are also factors affecting PE, which is dissimilar from our model. Although the role of tobacco is well established in arterial thrombosis, the evidence of its role in venous thromboembolism (VTE) as a distinct risk factor is less obvious and remains controversial (30–32). The possible reasons for the differences between our research and other studies are that smoking-attributable diseases or other predisposing factors are essential for smoking to convey a risk of VTE. It may also be due to our retrospective real-world research, where the data quality is not as good as prospective research, and there is sampling bias. However, the predictors included in our model differ from those in previous studies (33). Overall, three possible explanations for the discrepant results are as follows: (1) Our clinical research data platform did not have such indicators; (2) Indicators with a missing value rate higher than 20% were excluded; and (3) Following analysis, these indicators were not incorporated into the model.

This retrospective study suggested that a nomogram developed with clinical features and biomarkers to generate personalized evaluations of PE in respiratory department patients may distinguish target at high risk of PE. For example, a respiratory department patient’s total score is 321 points that corresponded to approximately 10.6% risk of PE. The proposed numerical model can assist clinicians in classifying respiratory department patients as either likely or unlikely to have PE, thereby reducing the number of unnecessary CTPA examinations. This model may be helpful for us to identify high-risk patients early, evaluate thrombosis, and implement active and individualized anticoagulation therapy.

The limitations of this study should be acknowledged. First, it is a retrospective study, and five indicators with a missing value rate exceeding 20% were excluded. Furthermore, the sample size was limited, and insufficient variables were recorded, potentially impacting the results. Finally, the data were collected from a single center and may not reflect a larger population.

In conclusion, we successfully developed a novel numerical model that can predict the risk of PE in respiratory department patients suspected of PE, which can not only appropriately select PE prevention strategies but also decrease unnecessary CTPA scans and their adverse effects.

## Data availability statement

The datasets presented in this study can be found in online repositories. The names of the repository/repositories and accession number(s) can be found in the article/Supplementary material.

## Ethics statement

The studies involving human participants were reviewed and approved by Medical Ethics Committee of Affiliated Dongyang Hospital of Wenzhou Medical University. Written informed consent for participation was not required for this study in accordance with the national legislation and the institutional requirements.

## Author contributions

FL and LW conceived and designed the research strategy. FL, MX, and JX wrote the manuscript text. CJ, ZJ, and MX collected the clinical data and participated in writing of the manuscript. FL, LW, and JX contributed to the analysis and interpretation of the data. All authors contributed to the article and approved the submitted version.

## Funding

This study was supported by Zhejiang Provincial Natural Science and Public Welfare Foundation of China under Grant no. LTGY23H200002 and Jinhua Science and Technology Foundation, Zhejiang Province, China (Grant nos. 2022-3-012 and 2022-4-269).

## Conflict of interest

The authors declare that the research was conducted in the absence of any commercial or financial relationships that could be construed as a potential conflict of interest.

## Publisher’s note

All claims expressed in this article are solely those of the authors and do not necessarily represent those of their affiliated organizations, or those of the publisher, the editors and the reviewers. Any product that may be evaluated in this article, or claim that may be made by its manufacturer, is not guaranteed or endorsed by the publisher.
