# Supplementary figures and images for: Developing a nomogram-based scoring model to estimate the risk of pulmonary embolism in respiratory department patients suspected of pulmonary embolisms
Source: Front Med (Lausanne). 2023 May 17;10:1164911. doi: 10.3389/fmed.2023.1164911 (PMC10229862; doi:10.3389/fmed.2023.1164911)

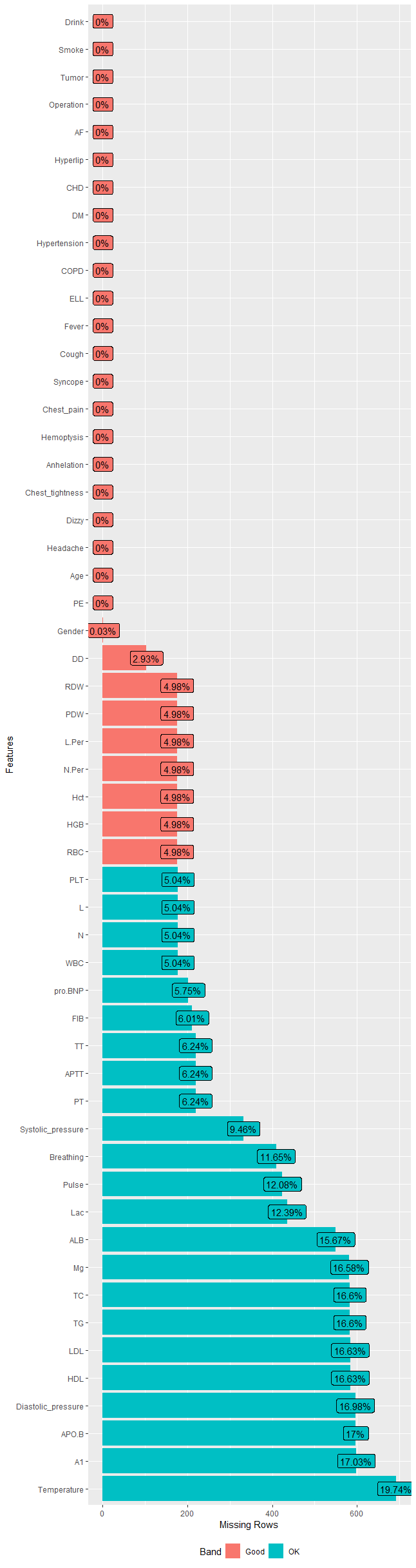

Supplement: Supplementary file 2 [file Image_1.TIFF]
